# Victimization by traditional bullying and cyberbullying and the combination of these among adolescents in 13 European and Asian countries

**DOI:** 10.1007/s00787-021-01779-6

**Published:** 2021-04-21

**Authors:** Roshan Chudal, Elina Tiiri, Anat Brunstein Klomek, Say How Ong, Sturla Fossum, Hitoshi Kaneko, Gerasimos Kolaitis, Sigita Lesinskiene, Liping Li, Mai Nguyen Huong, Samir Kumar Praharaj, Lauri Sillanmäki, Helena R. Slobodskaya, Jorge C. Srabstein, Tjhin Wiguna, Zahra Zamani, Andre Sourander, the Eurasian Child Mental Health Study (EACMHS) Group, Shahin Akhondzadeh, Daniel S. S. Fung, George Giannakopoulos, Meytal Grimland, Shoko Hamada, Emmi Heinonen, Raden Irawati Ismail, Praveen A. Jain, Avinash G. Kamath, Jerrine Z. N. Khong, Henriette Kyrrestad, Lotta Lempinen, Albert Prabowo Limawan, Maryam Mohseni, Ali Najafi, Minh Thanh Ngoc, Masayoshi Ogura, Zhekuan Peng, Tatiana O. Rippinen, Rini Sekartini, Nadezhda B. Semenova, Norbert Skokauskas, Yi Ren Tan, Kalliopi Triantafyllou, Phevous Zaravinos-Tsakos

**Affiliations:** 1 Department of Child Psychiatry, University of Turku, Turku, Finland; 2 Turku University Hospital, Turku, Finland; 3 Baruch Ivcher School of Psychology, Interdisciplinary Center (IDC), Herzlyia, Israel; 4 Department of Developmental Psychiatry, Institute of Mental Health, Singapore, Singapore; 5 The Regional Centre for Child and Youth Mental Health and Child Welfare, Faculty of Health Sciences, UiT the Arctic University of Norway, Tromsø, Norway; 6 Psychological Support and Research Center for Human Development, Nagoya University, Nagoya, Japan; 7 Department of Child Psychiatry, School of Medicine, National and Kapodistrian University of Athens, Aghia Sophia Children’s Hospital, Athens, Greece; 8 Faculty of Medicine, Institute of Clinical Medicine, Clinic of Psychiatry, Vilnius University, Vilnius, Lithuania; 9 Shantou University Medical College, Shantou, China; 10 Department of Psychiatry, Vietnam National Children’s Hospital, Hanoi, Vietnam; 11 Department of Psychiatry, Kasturba Medical College, Manipal, Manipal Academy of Higher Education, Manipal, Karnataka India; 12 Scientific Research Institute of Physiology and Basic Medicine, Novosibirsk State University, Novosibirsk, Russia; 13 Division of Psychiatry and Behavioral Sciences, Children’s National, Washington, DC USA; 14 Department of Psychiatry and Behavioral Sciences, School of Medicine, George Washington University, Washington, DC USA; 15 Department of Psychiatry, Faculty of Medicine, Universitas Indonesia-Dr. Cipto Mangunkusumo General Hospital, Jakarta, Indonesia; 16 Tehran University of Medical Sciences, Tehran, Iran; 17 INVEST Research Flagship, University of Turku, Turku, Finland

**Keywords:** Bullying, Cyberbullying, Victimization, Adolescent psychiatry, Cross-cultural

## Abstract

There has been a lack of studies on bullying in non-western low-income and middle-income countries. This study reported the prevalence of traditional victimization, cybervictimization, and the combination of these, in 13 European and Asian countries, and explored how psychiatric symptoms were associated with victimization. The data for this cross-sectional, school-based study of 21,688 adolescents aged 13–15 were collected from 2011 to 2017. The main outcomes were traditional and cybervictimization obtained from student self-reports. The Strengths and Difficulties Questionnaire was used to assess psychiatric symptoms. Generalized estimating equation and logistic regression models were used to estimate odds ratios (ORs) and 95% confidence intervals (95% CIs). The mean prevalence of any victimization was 28.9%, of traditional victimization only, this was 17.7%, and for cybervictimization only this was 5.1%. Cybervictimization occurred both independently, and in combination with, traditional victimization. The mean prevalence of combined victimization was 6.1%. The mean proportion of those who were cyberbullied only among those who were either cyberbullied only or bullied both traditionally and in cyber was 45.1%. The rates of prevalence varied widely between countries. In the total sample, those who experienced combined victimization, reported the highest internalizing symptoms (girls, OR 1.25, 95% CI 1.22–1.29; boys, OR 1.29, 95% CI 1.25–1.33). The study findings suggest that anti-bullying interventions should include mental health components and target both traditional and cyberbullying. Due to the overlap between these, targeting bullying should primarily focus on how to reduce bullying behavior rather than just focusing on where bullying takes place.

**Supplementary Information:**

The online version contains supplementary material available at 10.1007/s00787-021-01779-6.

## Introduction

Bullying is defined as intentional harmful behavior that involves an imbalance of power and results in repeated, aggressive behavior. This behavior can be physical, verbal, relational or damage someone’s property. Traditional bullying tends to occur in schools or neighborhoods, while cyberbullying is linked to technology, such as the Internet [1]. Both traditional [2–4] and cybervictimization [3–7] have been associated with adverse mental health effects among victims, and combined victimization was associated with even more severe psychiatric problems [4, 5]. Bullying can have a long-lasting negative impact on victims. These include anxiety and depression [8–10], suicidal behavior [11, 12], physical health problems, and socioeconomic disadvantages [10]. Victimization has also been associated with significantly reduced quality of life and financial losses [13].

Research on traditional bullying victimization has mostly been conducted in high-income western countries and the cross-national estimates of prevalence vary widely. A groundbreaking, cross-national bullying study was published in 2004 and comprised 25 high-income western countries and the prevalence of victimization varied from 5% in Sweden to 20% in Lithuania [14]. The mean prevalence rates for traditional victimization in two large surveys of 35 and 66 countries were 11% [15] and 37% [16], respectively. Other cross-cultural studies on traditional victimization also showed large variations in prevalence [15–20].

All cross-cultural studies on cybervictimization [6, 7, 21] and combined traditional and cybervictimization [3, 22–25] have been conducted in upper-middle and high-income countries. They reported wide variations in the estimates of the prevalence of cybervictimization across countries. This varied from 13% in Spain [6, 7] to 60% in Australia [21]. A large study on the victims of traditional and cyberbullying in 18 European countries found that traditional bullying ranged from 6% in Portugal to 20% in France and cyberbullying ranged from 3% in Portugal to 15% in Romania [23]. A study in 37 European and North American countries reported that 46% of those who had been cyberbullied had also reported traditional victimization, but there were wide variations across countries [22]. There have been ongoing discussions about whether traditional and cyberbullying are distinct entities or different presentations of the same phenomenon [26, 27]. While some studies have suggested that there are differences between them [28, 29], others have suggested that cyberbullying is just another manifestation of bullying [4, 26]. This is an important issue when planning anti-bullying interventions.

The major limitations of the existing cross-cultural studies have included the lack of studies that have focused on different types of victimization, particularly the overlap between traditional victimization and cybervictimization in countries with different socioeconomic development. Another limitation has been the lack of cross-cultural studies on the associations between psychiatric symptoms and victimization in these countries. There have been limited opportunities to compare countries due to different study methods, including the definitions of bullying. The lack of a well-established definition of cyberbullying has been a particular issue [30]. The present study broadens the existing research by describing victimization in 13 countries with different economic profiles. It used the same study method in all of the countries, focused on different types of victimization and assessed the associations between psychiatric symptoms and victimization. In addition to describing differences in victimization across countries, we also explored within-country differences and associations between victimization and the availability of anti-bullying interventions and the level of development of the country.

This study had four aims. The first was to report cross-cultural comparisons of the prevalence of traditional victimization, cybervictimization, and a combination of these, among adolescents in 13 Asian and European countries with lower-middle, upper-middle or high income. The second aim was to report the extent to which traditional victimization and cybervictimization overlapped in this multi-country sample. The third aim was to examine the associations between internalizing and externalizing symptoms and victimization. Fourth, we aimed to shed some light on whether there were any variations in the probability of victimization between schools in different countries, and whether victimization was associated with the availability of anti-bullying interventions and the development of the country. This was the first cross-cultural study to examine the prevalence of these categories of victimization, the association between psychiatric symptoms and victimization and the overlap of traditional and cybervictimization in countries with such differences in socioeconomic development.

## Methods

This study is part of the Eurasian Child Mental Health Study (EACMHS) and included eight Asian countries and five European countries: China, Finland, Greece, India, Indonesia, Iran, Israel, Japan, Lithuania, Norway, Russia, Singapore and Vietnam. The EACMHS aims to conduct cross-cultural, multi-site research on the wellbeing and mental health of children and adolescents and it includes child and adolescent mental health experts in the participating countries [31–33].

### Sample

Our research was based on a survey of 28,427 adolescents and the data were collected between 2011 and 2017. The response rates varied from 51.7% in Indonesia to 97.1% in Iran, with a median of 88.9%. A subsample of 21,688 adolescents, aged 13–15 years, from 200 schools, were included in this study to increase the comparability of the data across countries. This was because there were variations in the age ranges in the total samples across countries. Each country selected their own schools, so that they provided a mix of both urban and rural schools, as well as public and privately funded schools. The characteristics of the study sample and the survey year in each country are presented in Table 1.

**Table 1:** Characteristics of the 21,688 adolescents aged 13–15 from the 13 countries that were included in this analysis of the Eurasian Child Mental Health Study

Country	Survey year	Total sample	Subsample								Schools
				Girls	Boys	Urban residence	Rural residence	Public school	Private school	Age	
		*n*	*n*	*n* (%)	*n* (%)	*n* (%)	*n* (%)	*n* (%)	*n* (%)	Mean (SD)	*N*
Japan	2011	1842	1828	943 (51.6)	885 (48.4)	833 (45.5)	998 (54.5)	1831 (100)	0 (0)	13.9 (0.3)	17
Greece	2016	1581	1040	556 (53.5)	**484 (46.5)**	750 (72.1)	290 (27.9)	1040 (100)	0 (0)	13.6 (0.6)	14
Norway	2017	2019	1900	946 (49.8)	954 (50.2)	1611 (84.8)	289 (15.2)	1742 (99.4)	10 (0.6)	13.9 (0.8)	45
China	2016	2659	2119	1040 (49.1)	1079 (50.9)	819 (36.8)	1408 (63.2)	1779 (79.9)	448 (20.1)	13.8 (0.8)	10
India	2016	2016	1672	864 (51.7)	808 (48.3)	1420 (84.9)	252 (15.1)	209 (12.5)	1463 (87.5)	13.6 (0.7)	11
Finland	2014	3422	2982	1493 (50.1)	1489 (49.9)	2686 (89.9)	301 (10.1)	2988 (100)	0 (0)	14.1 (0.8)	13
Singapore	2014	3319	2165	1103 (51.0)	1062 (49.1)	2165 (100)	0 (0)	2165 (100)	0 (0)	14.0 (0.8)	24
Vietnam	2016	1118	946	484 (51.2)	462 (48.8)	946 (100)	0 (0)	946 (100)	0 (0)	13.9 (0.8)	3
Israel	2014	2188	1277	698 (54.7)	**579 (45.3)**	1101 (100)	0 (0)	1246 (97.4)	33 (2.6)	14.0 (0.8)	10
Iran	2016	1456	1178	557 (47.3)	621 (52.7)	1178 (100)	0 (0)	1036 (88.0)	142 (12.1)	14.3 (0.8)	16
Lithuania	2016	3837	2507	1256 (50.1)	1251 (49.9)	1353 (53.8)	1162 (46.2)	2515 (100)	0 (0)	14.1 (0.8)	17
Russia	2015	1580	1051	546 (52.0)	505 (48.1)	1051 (100)	0 (0)	1051 (100)	0 (0)	14.1 (0.8)	20
Indonesia	2016	1390	1023	542 (53.0)	481 (47.0)	1024 (100)	0 (0)	656 (64.1)	368 (35.9)	13.5 (0.6)	5
Total	2011–2017	28,427	21,688	11,028 (50.9)	**10,660 (49.2)**	16,937 (78.3)	4700 (21.7)	19,204 (88.6)	2464 (11.4)	13.9 (0.8)	200

Chi-square test for equal proportions was used to analyze sex distribution. Bold type indicates statistical significance of at least *p* < 0.05. *SD* standard deviation

### Questionnaire and procedure

The survey comprised a self-administered questionnaire that was based on one that was previously used in surveys of adolescents in Finland [5, 34]. The questionnaires were translated into the local language and back translated in each country to ensure uniformity. All students who were present in the class at the time of the survey were invited to participate and filled in the questionnaires anonymously during school lessons. The questionnaires were then collected in a confidential manner and returned to the researchers by the teachers. The questionnaire was completed electronically in Norway and Singapore.

### Measures

The demographic information included age and sex. Adolescents were asked about their experiences of traditional victimization and cybervictimization. The following definition of traditional bullying was provided in the questionnaire: “A student is getting bullied, if another student or a group of students repeatedly treats him/her negatively or in an insulting manner. It is difficult for the bullied student to defend himself/herself. Bullying can be intermittent or continuous. Bullying can be verbal (e.g. calling names, threatening), physical (e.g. hitting, pushing) or psychological (e.g. spreading rumors, avoiding, excluding). Continuous nasty or insulting teasing is also bullying.” Cyberbullying was defined as: “Repeated mocking on the Internet, bullying via emails or text messages or spreading insulting material about another person on the Internet.” The students were asked how often they had been bullied at school or outside school or cyberbullied in the past 6 months. The same four-point response scale was used throughout the questionnaire. The options were never, less than once a week, more than once a week and almost every day. We combined the responses into binary outcomes: no for never and yes for the other responses.

Psychiatric symptoms were assessed with a self-report version of the Strengths and Difficulties Questionnaire (SDQ). The validity and reliability of the SDQ have been found to be satisfactory [35, 36]. The SDQ has been translated into more than 80 languages [37] and it has been widely used in a number of cross-cultural studies [38–40]. The SDQ consists of 25 items that are divided into five subscales: emotional symptoms, conduct problems, hyperactivity, peer problems and prosocial [41]. These can be combined to provide an internalizing scale, which brings together the emotional and peer problems subscales, and an externalizing scale, which comprises the conduct problems and hyperactivity subscales. The internalizing and externalizing scales have shown good validity with respect to clinical disorders. However, the discriminant validity has been reported to be poorer between the individual emotional symptoms and peer problems subscales and the conduct problems, hyperactivity and prosocial subscales. This has been a particular issue when cohorts have recorded low scores for the individual scales. As a result, researchers have been advised to use the combined internalizing and externalizing scales when analyzing low-risk samples [42]. Our study samples were drawn from the general populations in each country and that is why we used the internalizing and externalizing scales, as these samples were regarded as low-risk. The question on bullying was excluded from the analyses.

The study also explored the associations between victimization and the availability of focused anti-bullying interventions and the development of the country (see Online Resource). The development of the country was assessed using the Human Development Index (HDI), which measures key dimensions of human development, including having a long and healthy life, being knowledgeable and having a decent standard of living [43]. Information from the child and adolescent mental health experts from each country participating in the EACMHS was used to classify the countries based on the availability of anti-bullying interventions. Countries were categorized as having anti-bullying interventions if bullying was regarded as a national priority and focused anti-bullying interventions were available at the participating schools before the survey was conducted.

### Statistical analysis

The responses from all countries were pooled together to create various descriptive statistics for the sample. Sex × country interaction for bullying victimization was found significant (*p* < 0.001). Therefore, further analyses were conducted separately for each sex. Generalized estimating equation (GEE) models were conducted to estimate the odds ratios (ORs) and 95% confidence intervals (95% CIs) for the odds of various types of victimization in the different countries. The reference category that was chosen a priori was the country with the lowest prevalence of victimization and this was Japan. Unadjusted ORs and 95% CIs were estimated and school-wise clusters were included in these statistical models. Adjustments were made for the age of the participants.

We analyzed the association between psychiatric symptoms and the types of victimization for the total sample. The outcome variable was victimization and the explanatory variables were the continuous SDQ internalizing and externalizing scales. This was not carried out by country because the sample sizes were too small for some of the countries. Sex × externalizing scale interaction for victimization was found significant (*p* = 0.0013). Therefore, the analyses were carried out separately for each sex. The generalized linear mixed model (GLMM) with school-wise random intercepts was used to estimate the ORs and 95% CIs. The reference groups were those who were not victimized. The data were adjusted for age and externalizing symptoms when we analyzed the internalizing symptoms and vice-versa. Adjustments were also made for country. We also assessed the association between psychiatric symptoms and any victimization by country. Interactions between sex and the SDQ internalizing and externalizing scales for any victimization were tested by country. If the analysis of that country was not significant, that particular sample was pooled for further analyses. However, if there were significant interactions in that country, further analyses were conducted separately for each sex. We used GEE models to estimate the ORs and 95% CIs and school-wise clusters were included in the models. The data were adjusted for age and externalizing symptoms when we analyzed the internalizing symptoms and vice-versa. In addition, the data for the pooled countries were adjusted for sex. The reference categories were the subjects who were not victimized in each country.

In additional analyses, within-country differences in victimization and the association between victimization and the availability of focused anti-bullying interventions and the development of the country were explored. To assess within-country variations, model-generated, age-adjusted predicted probabilities for any victimization were estimated by sex for each school in each country. When the association between victimization and the availability of anti-bullying interventions and the development of the country was explored, a composite variable was used to estimate ORs using the GEE. The countries were grouped based on their HDI ranks and whether anti-bullying interventions were available in their schools. These two factors were then combined into three composite variables: very high HDI countries with anti-bullying programs, very high HDI countries without such programs and high/medium HDI countries with no programs. For further details on the additional analyses, see Online Resource.

Two-sided *p* values of less than 0.05 were considered statistically significant, except for the interactions for which the threshold was 0.1. The statistical analyses were conducted using SAS 9.4 for Windows (SAS Institute Inc. Cary, NC, USA, 2012).

## Results

There were responses from 21,688 adolescents in 13 Asian and European countries and these varied from 946 in Vietnam to 2982 in Finland. They were aged 13.0–15.0 years (M 13.9, SD 0.8). The prevalence of victimization by country, and the type of victimization, is shown in Table 2. It is also shown by country, and frequency of victimization, in Table S1 (see Online Resource). The prevalence of any victimization was 28.1% for the whole sample, traditional victimization only was 17.5%, cybervictimization only was 4.7% and combined victimization was 5.8%. The mean of the prevalence for any victimization in the 13 countries was 28.9%, it was 17.7% for traditional victimization only, 5.1% for cybervictimization only and 6.1% for combined victimization. These rates represent the means of the 13 country-wise prevalence rates in each victimization category.

**Table 2:** Prevalence of bullying victimization by country

Country	Participants	Victimization					
		None	Any	Traditional only	Cyber only	Combined	Cyber only/all cyber^a^ %
	*n*	*n* (%)	*n* (%)	*n* (%)	*n* (%)	*n* (%)	
Japan	1768	1484 (83.9)	284 (16.1)	245 (13.9)	17 (1.0)	22 (1.2)	43.6
Greece	1037	869 (83.8)	168 (16.2)	119 (11.5)	28 (2.7)	21 (2.0)	57.1
Norway	1900	1524 (80.2)	376 (19.8)	143 (7.5)	123 (6.5)	110 (5.8)	52.8
China	2132	1667 (78.2)	465 (21.8)	275 (12.9)	119 (5.6)	71 (3.3)	62.6
India	1526	1172 (76.8)	354 (23.2)	301 (19.7)	19 (1.3)	34 (2.2)	35.8
Finland	2895	2103 (72.6)	792 (27.4)	544 (18.8)	100 (3.5)	148 (5.1)	40.3
Singapore	2157	1536 (71.2)	621 (28.8)	346 (16.0)	83 (3.9)	192 (8.9)	30.2
Vietnam	945	655 (69.3)	290 (30.7)	220 (23.3)	26 (2.8)	44 (4.7)	37.1
Israel	1265	871 (64.6)	448 (35.4)	260 (20.6)	81 (6.4)	107 (8.5)	43.1
Iran	1146	730 (63.7)	416 (38.7)	214 (18.7)	106 (9.3)	96 (8.4)	52.5
Lithuania	2388	1502 (62.9)	886 (37.1)	618 (25.9)	110 (4.6)	158 (6.6)	41.0
Russia	1021	623 (61.0)	398 (39.0)	181 (17.7)	126 (12.3)	91 (8.9)	58.1
Indonesia	1024	574 (56.1)	450 (43.9)	241 (23.5)	67 (6.5)	142 (13.9)	32.1
Total sample	21,204	15,256 (72.0)	5948 (28.1)	3707 (17.5)	1005 (4.7)	1236 (5.8)	44.8

Any refers to traditional victimization, cyberbullying victimization or both of these. Combined refers to both traditional victimization and cyberbullying victimization

^a^The proportion of those who were just exposed to cyberbullying as a percentage of the combined cyberbullying and traditional bullying category

**Fig. 1:**
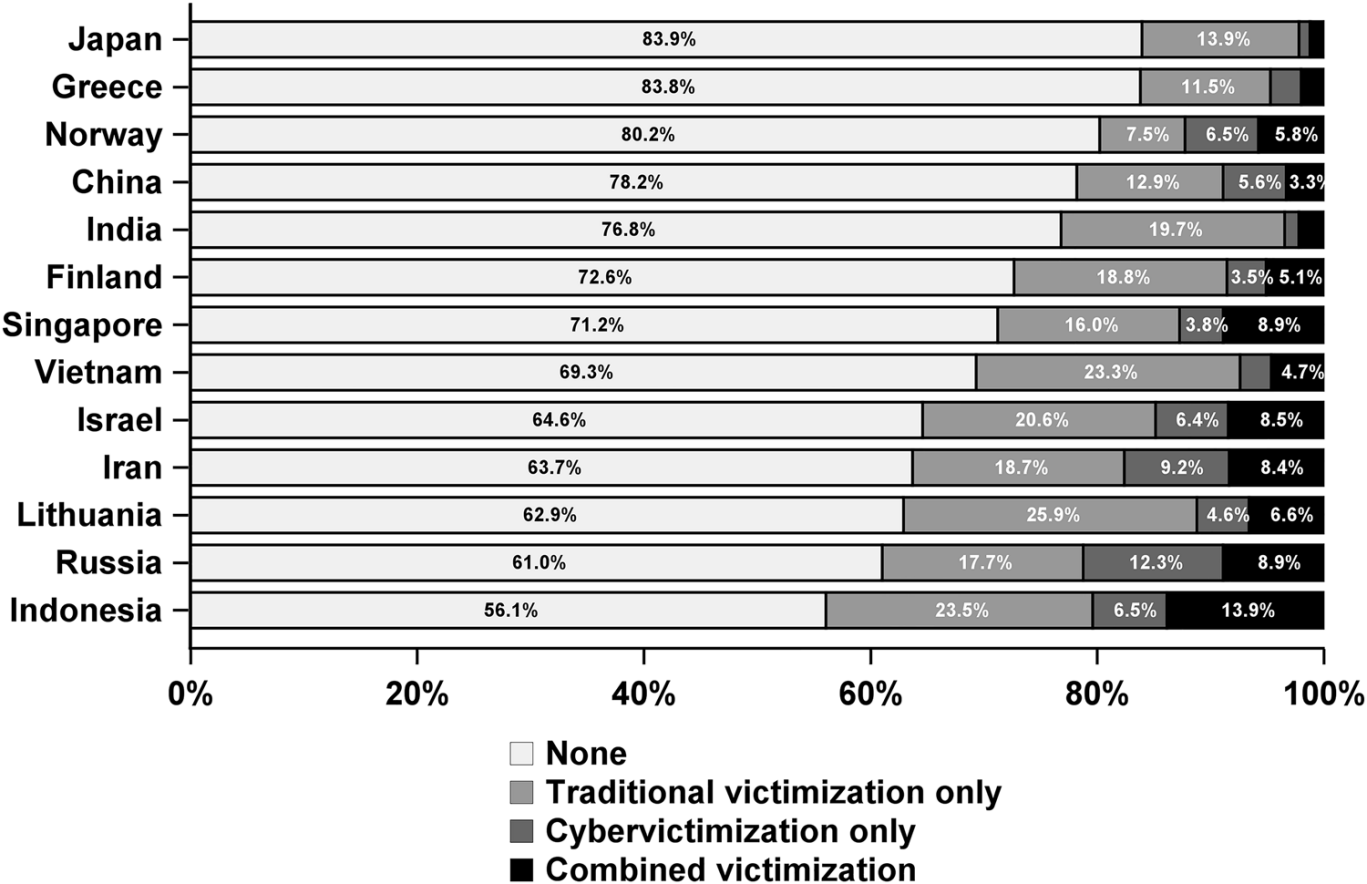
Distribution of different types of victimization in each country

The prevalence of any victimization varied from 16.1% in Japan to 43.9% in Indonesia (Table 2, Fig. 1). Traditional victimization only varied from 7.5% in Norway to 25.9% in Lithuania and cybervictimization only ranged from 1.0% in Japan to 12.3% in Russia. Combined victimization ranged from 1.2% in Japan to 13.9% in Indonesia. Figure 1 shows the distribution of different types of victimization in each country. The proportion of those who were cyberbullied only among those who were either cyberbullied only or bullied both traditionally and in cyber was calculated. This varied from 30.2% in Singapore to 62.6% in China, with a mean of 45.1% (Table 2).

Table 3 shows the odds of any victimization in the different countries, compared to Japan, which was the reference country. Girls in all countries, except Greece, China and India, had increased odds of any victimization and the greatest odds were in Indonesia (OR 3.98, 95% CI 2.32–6.83). Boys in all countries except Greece and Norway had increased odds of any victimization and the greatest odds were also seen in Indonesia (OR 4.93, 95% CI 2.94–8.27).

**Table 3:** Odds for any victimization among girls and boys by country

	Girls			Boys		
Country	Total *n*	Victimized *n* (%)	OR (95% CI)	Total *n*	Victimized *n* (%)	OR (95% CI)
Japan	925	149 (16.1)	1	846	141 (16.7)	1
Greece	556	81 (14.6)	0.90 (0.61 − 1.33)	482	88 (18.3)	1.12 (0.76 − 1.66)
Norway	946	213 (22.5)	**1.56 (1.08 − 2.24)**	954	163 (17.1)	1.09 (0.81 − 1.45)
China	1012	189 (18.7)	1.19 (0.85 − 1.67)	1040	275 (26.4)	**1.89 (1.46 − 2.45)**
India	803	146 (18.2)	1.14 (0.66 − 1.98)	747	232 (31.1)	**2.43 (1.57 − 3.76)**
Finland	1471	419 (28.5)	**2.13 (1.52 − 2.96)**	1426	381 (26.7)	**2.02 (1.52 − 2.69)**
Singapore	1102	308 (28.0)	**2.01 (1.37 − 2.95)**	1058	316 (29.9)	**2.32 (1.79 − 3.00)**
Vietnam	483	125 (25.9)	**1.81 (1.31 − 2.50)**	462	165 (35.7)	**3.06 (2.04 − 4.61)**
Israel	692	229 (33.1)	**2.34 (1.57 − 3.49)**	573	222 (38.7)	**3.12 (2.37 − 4.12)**
Iran	533	162 (30.4)	**2.36 (1.67 − 3.32)**	621	262 (42.2)	**4.07 (3.10 − 5.34)**
Lithuania	1222	467 (38.2)	**3.39 (2.38 − 4.81)**	1198	457 (38.2)	**3.44 (2.58 − 4.60)**
Russia	543	203 (37.4)	**3.20 (2.24 − 4.58)**	480	197 (41.0)	**3.79 (2.60 − 5.53)**
Indonesia	542	230 (42.4)	**3.98 (2.32 − 6.83)**	481	220 (45.7)	**4.93 (2.94 − 8.27)**

GEE model with school-wise clusters included. Adjusted for age. Differences in the numbers of participants between tables are due to missing information. Bold type indicates statistical significance of at least *p* < 0.05

*OR* odds ratio

Table 4 shows the association between internalizing and externalizing symptoms and victimization in the total sample. We found that both internalizing and externalizing symptoms were significantly associated with traditional victimization only, cybervictimization only, and the combination of these, in girls and boys in the total sample, when they were compared to those who were not victimized.

**Table 4:** Odds for internalizing and externalizing symptoms in those adolescents who were victims of traditional bullying only, cyberbullying only or the combination of these, with their corresponding *p* values

	Internalizing				Externalizing			
	Girls		Boys		Girls		Boys	
	OR (95% CI)	*p* value	OR (95% CI)	*p* value	OR (95% CI)	*p* value	OR (95% CI)	*p* value
Traditional only vs. none	1.21 (1.19–1.23)	< 0.0001	1.19 (1.17–1.22)	< 0.0001	1.05 (1.03–1.08)	< 0.0001	1.04 (1.02–1.06)	< 0.0001
Cyber only vs. none	1.09 (1.06–1.13)	< 0.0001	1.07 (1.03–1.11)	0.0002	1.14 (1.11–1.18)	< 0.0001	1.08 (1.04–1.11)	< 0.0001
Combined vs. none	1.25 (1.22–1.29)	< 0.0001	1.29 (1.25–1.33)	< 0.0001	1.17 (1.14–1.21)	< 0.0001	1.10 (1.06–1.13)	< 0.0001
Combined vs. traditional only	1.05 (1.02–1.08)	0.0021	1.09 (1.06–1.13)	< 0.0001	1.12 (1.09–1.16)	< 0.0001	1.06 (1.03–1.10)	0.0003
Combined vs. cyber only	1.16 (1.11–1.20)	< 0.0001	1.20 (1.15–1.26)	< 0.0001	1.03 (0.99–1.08)	0.1079	1.04 (0.99–1.09)	0.0906
Traditional only vs. cyber only	1.10 (1.06–1.14)	< 0.0001	1.11 (1.06–1.16)	< 0.0001	0.93 (0.88–0.95)	< 0.0001	0.98 (0.94 – 1.02)	0.2392

Sex × internalizing scale for victimization *p* value was 0.3936 and sex × externalizing scale for victimization *p* value was 0.0013. GLMM model with school-wise random intercepts. The odds ratios have been estimated for a one-point rise in the symptom scales. Internalizing symptoms were adjusted for age, country and the externalizing SDQ scale. Externalizing symptoms were adjusted for age, country and the internalizing SDQ scale. *OR* odds ratio, *GLMM*, generalized linear mixed model, *SDQ* the Strengths and Difficulties Questionnaire

When different victimization groups were compared, combined victimization had a significantly higher association with internalizing symptoms in girls and boys than traditional victimization only (OR 1.05, 95% CI 1.02–1.08; OR 1.09, 95% CI 1.06–1.13, respectively) or cybervictimization only (OR 1.16, 95% CI 1.11–1.20; OR 1.20, 95% CI 1.15–1.26, respectively). Similarly, combined victimization had a significantly stronger association with externalizing symptoms, when compared with traditional victimization only in girls and boys (OR 1.12, 95% CI 1.09–1.16; OR 1.06, 95% CI 1.03–1.10, respectively), but not when compared with cybervictimization only (OR 1.03, 95% CI 0.99–1.08; OR 1.04, 95% CI 0.99–1.09, respectively). Finally, when traditional victimization only and cybervictimization only were compared, both the girls and boys in the traditional victimization only groups reported higher level of internalizing symptoms (OR 1.10, 95% CI 1.06–1.14; OR 1.11, 95% CI 1.06–1.16, respectively) (Table 4).

Table 5 shows that internalizing and externalizing symptoms were significantly associated with any victimization among adolescents in most countries. The associations between internalizing symptoms and any victimization did not reach statistical significance in boys in Greece and girls in Indonesia. Nor did the associations between externalizing symptoms and any victimization in boys and girls in Japan and boys in Greece, Norway and Lithuania.

**Table 5:** Odds for internalizing and externalizing symptoms in those adolescents who were victims of any bullying, with their corresponding *p* values. The reference groups for each country were those who were not victims of bullying

	Internalizing		Externalizing	
Country	OR (95% CI)	*p* value	OR (95% CI)	*p* value
Japan	1.26 (1.23 − 1.29)	< 0.0001	1.05 (0.999 − 1.10)	0.0579
Greece				
Girls	1.21 (1.13 − 1.31)	< 0.0001	1.15 (1.07 − 1.24)	0.0002
Boys	1.10 (0.99 − 1.22)	0.0773	1.08 (0.98 − 1.19)	0.1274
Norway				
Girls	1.23 (1.16 − 1.30)	< 0.0001	1.17 (1.09 − 1.26)	< 0.0001
Boys	1.20 (1.15 − 1.26)	< 0.0001	1.04 (0.99 − 1.08)	0.1066
China	1.21 (1.13 − 1.28)	< 0.0001	1.10 (1.06 − 1.13)	< 0.0001
India				
Girls	1.19 (1.12 − 1.25)	< 0.0001	1.13 (1.03 − 1.23)	0.0071
Boys	1.09 (1.02 − 1.17)	0.0096	1.12 (1.03 − 1.22)	0.0062
Finland				
Girls	1.29 (1.22 − 1.35)	< 0.0001	1.11 (1.05 − 1.17)	0.0003
Boys	1.31 (1.23 − 1.39)	< 0.0001	1.05 (1.02 − 1.08)	0.0003
Singapore	1.21 (1.17 − 1.25)	< 0.0001	1.06 (1.03 − 1.10)	0.0005
Vietnam	1.08 (1.05 − 1.12)	0.0028	1.06 (1.02 − 1.10)	0.0022
Israel	1.20 (1.16 − 1.24)	< 0.0001	1.05 (1.02 − 1.09)	0.0041
Iran				
Girls	1.11 (1.05–1.18)	0.0003	1.11 (1.03–1.21)	0.0099
Boys	1.08 (1.01–1.16)	0.0191	1.14 (1.02–1.28)	0.0212
Lithuania				
Girls	1.27 (1.19 − 1.35)	< 0.0001	1.08 (1.03 − 1.13)	0.0009
Boys	1.24 (1.18 − 1.30)	< 0.0001	1.01 (0.96 − 1.05)	0.8189
Russia	1.14 (1.09 − 1.19)	< 0.0001	1.10 (1.06 − 1.13)	< 0.0001
Indonesia				
Girls	0.97 (0.94 − 1.01)	0.0940	1.13 (1.06 − 1.20)	0.0001
Boys	1.05 (1.01 − 1.09)	0.0180	1.08 (1.02 − 1.14)	0.0055

The results are shown for girls and boys separately if there were significant interactions (*p* < 0.1) between sex and internalizing or externalizing symptoms for any victimization. In Iran, the model did not converge and the results are shown separately for girls and boys, even though the interactions did not reach statistical significance. Significant interactions were found in Greece (*p* = 0.0759), Norway (*p* = 0.0537), India (*p* = 0.0821) and Indonesia (*p* = 0.0579) between sex and the internalizing scale for victimization. Significant interactions were found in Norway (*p* = 0.0021), Finland (*p* = 0.0634) and Lithuania (*p* = 0.0961) for the sex and the externalizing scale for victimization. GEE model with school-wise clusters included. The odds ratios have been estimated for a one-point rise in the symptom scales. Internalizing symptoms were adjusted for age and the externalizing SDQ scale. Externalizing symptoms were adjusted for age and the internalizing SDQ scale. Adjustment was also made for sex when the pooled sample was analyzed. *OR* odds ratio, *SDQ* the Strengths and Difficulties Questionnaire

Tables S2 and S3 show the prevalence and odds of the different types of bullying by sex. Table S2 shows the greatest prevalence of victimization in girls by country. For traditional victimization only it was highest in Lithuania (26.0%) for cybervictimization only it was Iran (11.4%) and for combined victimization it was Indonesia (14.9%). Table S3 shows that for boys it was highest in Indonesia for traditional victimization only (27.0%), Russia for cybervictimization only (17.1%) and Indonesia for combined victimization (12.7%).

In additional analyses (see Online Resource), we explored within-country differences in victimization. Fig. S1 shows the predicted probabilities of any victimization by school and country by sex. For girls, the range in predicted probabilities of victimization between schools was smallest in Vietnam and largest in Japan. For boys, the range was smallest in China and largest in India. The correlation in GEE-models was rather low, both within schools (0.037) and within countries (0.027). This indicates that there were variations in victimization in both, but these were smaller within countries than schools. We also compared very high HDI countries with anti-bullying programs with very high HDI countries and high/medium countries without such programs. In both cases, not having a program was associated with increased odds of traditional victimization only, cybervictimization only and combined victimization in boys. In very high HDI countries, it was also associated with combined victimization in girls, but the same results were not found in high/medium HDI countries (Table S4).

## Discussion

This study had three key findings. The first was that both traditional and cybervictimization were a global problem. Second, cybervictimization occurred both independently and combined with traditional victimization and the proportion of those who were cyberbullied only was considerable in all of the participating countries. Third, both internalizing and externalizing symptoms were associated with victimization in most countries and those individuals who experienced combined victimization reported the highest levels of internalizing symptoms. Fourth, we explored variations in victimization and found that these were smaller within countries than within schools. Furthermore, adolescents in countries with a very high HDI and anti-bullying interventions were less likely to be victimized than adolescents, mostly boys, living in very high or high/medium HDI countries with no such programs.

This was the first study to report concurrent traditional and cybervictimization in a large sample of adolescents in lower-middle-, upper-middle-, and high-income countries. The prevalence of any victimization ranged from 16.1% in Japan to 43.9% in Indonesia. Traditional victimization only was the most common type (17.7%), followed by combined victimization (6.1%) and cybervictimization only (5.1%). We found wide variations in bullying between countries, regardless of their HDI ranks. It is possible that the availability of smartphones and Internet access by adolescents could have varied across countries and influenced the findings on cybervictimization. Cultural factors may have affected our findings, despite the uniform definitions of bullying and cyberbullying that were provided in the questionnaire. For example, there may have been cultural differences in which incidents were regarded as bullying [44]. To date, most research on bullying has been conducted in western countries. The wide variations in the prevalence rates between the countries in our study emphasize the importance of cross-cultural research on different types of bullying.

The results show that cybervictimization occurred both independently, and in combination with, traditional victimization, with being exposed to just cyberbullying accounting for 45.1% (range 30.2% to 62.6%). There have been discussions about whether traditional and cyberbullying are distinct phenomena [26, 27]. Some studies have suggested cyberbullying is just another way to bully those already bullied traditionally and very few new victims are created [4, 26]. However, comparable rates of cybervictimization have been found among home-schooled adolescents and those who attended school, suggesting that cyberbullying was not necessarily just an extension of traditional school bullying [45]. Our study found that cybervictimization was a diverse phenomenon, which occurred both independently and together with traditional victimization. Despite the fact that we provided uniform definitions of traditional and cyberbullying in the questionnaire, the prevalence of cybervictimization only was different in the participating countries and ranged from 1.0% in Japan to 12.3% in Russia. Thus, cyberbullying may create new victims and may also aggravate problems faced by those who are already victims of traditional bullying and who also become cyberbullied. There is variation in these rates among countries. This has important implications for anti-bullying actions, as it suggests that we need to focus on reducing all kinds of bullying behavior and enhancing prosocial interaction rather than primarily focusing on where the bullying takes place [30]. Greater understanding of the differences in victimization between sexes would also help to enhance anti-bullying efforts. A previous study found that various components of anti-bullying programs had different effects on girls and boys [46]. Studies have reported that physical bullying was less typical among girls [47] but girls were more likely than boys to be victims of relational and cyberbullying [4]. Physical and verbal bullying are more obvious and are more likely to be tackled. These variations may have an impact on how effective anti-bullying interventions are on different sexes and cultures [48]. Although preventive programs that have been designed to tackle school bullying [49] and cyberbullying [50] have been effective in western countries, we still lack knowledge on their effectiveness especially in low-income and middle-income countries [51].

Our third main finding was that victimization was associated with internalizing and externalizing symptoms in most countries. We also found that those who experienced combined victimization reported the highest internalizing symptoms. Cyberbullying can reach the victim at any place and time, which means diminished possibilities to escape bullying outside school. Furthermore, the victim may not know the identity of the bully in cyber context [52]. It is possible that these features of cyberbullying, when it occurs in combination with traditional bullying, predispose the victims to more severe outcomes. Health services that treat adolescents need to consider victimization as an indicator of comorbid psychiatric symptoms and assess them for both victimization and psychopathology [5]. Treatment and follow-up visits should be provided, because both internalizing [53] and externalizing [54] symptoms have been found to have a bi-directional relationship with bullying victimization. This may help to create a vicious circle, in which victimization is associated with higher levels of psychiatric symptoms and those symptoms perpetuate the victimization [53]. Longitudinal population-based studies have shown a strong association between childhood victimization and psychiatric disorders in adulthood [9, 55] and the association was stronger in individuals who had psychiatric symptoms in childhood [55]. Even after controlling for childhood psychiatric symptoms, victimization was associated with anxiety [9] and depression [56] in adulthood. It has been stated that reducing any involvement in bullying could reduce mental health problems up to adulthood [57]. However, as emphasized previously, research on the adverse effects of bullying has concentrated on western countries. Our study focused on 13 Asian and European countries with different socioeconomic development and indicated that, in most countries, victimization was an indicator of mental health symptoms. Although we lack knowledge on the effectiveness of anti-bullying programs in low-income and middle-income countries [51], our socioecological understanding of bullying prevention includes measures that promote good mental health [58]. Our findings emphasize the importance of providing mental health promotion as part of bullying prevention programs. This could include providing psychoeducation to students, their parents and teachers, so that they have the skills they need to enhance their mental resilience, cope with life and seek help when they need it. The strong association between combined victimization and psychiatric symptoms found by this study indicates that both traditional and cyberbullying need to be tackled.

Our fourth finding was that variations in victimization were smaller within countries than within schools. Previous cross-cultural studies have mainly concentrated on differences between countries. However, a study of 18 European countries found that differences within countries were smaller than differences between them [23]. Two studies have reported that economic inequality at a national level was associated with increased victimization [15, 59]. These findings, and the differences found between the schools in our study, highlight the importance of how individual schools manage bullying. It has been reported that the positive effects of anti-bullying programs observed in randomized controlled trials tend to decline during real-life implementation [60]. Positive school environments, which are fair and trustworthy and make pupils feel connected and safe, have been reported to provide protective factors against bullying [61] and these may have positive effects when developing and implementing anti-bullying actions.

We also piloted in exploring the association between victimization and the availability of focused anti-bullying interventions and the development of the country. The results must be interpreted with caution, due to the cross-sectional design, the low number of countries that were assessed in each group and the possible factors that may have affected the prevalence of bullying in the participating countries, such as media coverage on the subject. However, in general, adolescents in countries with a very high HDI and anti-bullying interventions were less likely to be victimized than adolescents, mostly boys, who were living in very high or high/medium HDI countries with no such programs. Previously, some studies have assessed whether there was any association between economic inequality and bullying victimization. One study of 35 countries failed to show any link between the economic level of the country and victimization [15]. Our findings support the positive role of anti-bullying interventions and indicate the importance of implementing these in countries where they are not currently available. Further cross-cultural research is warranted to examine the effectiveness, feasibility, and implementation of antibullying programs in countries with different levels of development.

The strength of our study included data on the victimization of adolescents in 13 countries at different levels of socioeconomic development. We also used the same questions and the same definitions of bullying and cyberbullying in each country. However, some limitations need to be borne in mind. First, the study was conducted in certain regions of the 13 countries and the findings may not represent the countries as a whole. We aimed to select public and private schools in both urban and rural locations. However, the representativeness of the study may have been affected by wide within-country differences in locations such as China and India. In India, for example, the sample largely consisted of private schools and this should be considered when interpreting those findings. Second, most of the data were collected from 2014 to 2017. The only exception was Japan, where the data were collected in 2011. This may have affected the comparability of the findings across countries, as the technology that was available may have been different in the various years, for example. Third, we did not have data on Internet accessibility or the availability of smartphones among. That meant that we could not control for these when we assessed cybervictimization. Fourth, the study population in various countries may have understood bullying and cyberbullying differently, for example due to cultural differences, and the true rates may have been misreported in some countries. Fifth, the present study addressed traditional and cyberbullying, but lacked information on other types of bullying, like sibling bullying, which has been reported to be common [62], as well as bullying in the workplace [63] as most of the countries surveyed in this study, with the exception of Lithuania and Russia, established 15 years of age (or 14 a in the case of India) as the minimum age for entry into work or employment [64, 65]. Sixth, when we analyzed the association between psychiatric symptoms and victimization, it was not meaningful to conduct analyses by country because of small number of cases especially in cybervictimization groups. Seventh, in the additional analyses, the 13 countries were stratified into HDI categories when we looked at the availability of anti-bullying interventions. The number of countries in each group was small, which limits the generalizability of the associations. It is possible that other factors like public awareness of the harmful effects of bullying and media coverage on the subject and awareness of and access to anti-bullying resources varied from country to country. Lastly, the cross-sectional study design meant that the study was purely observational and no causal inference can be drawn from the findings.

## Conclusion

This study adds to the literature on bullying victimization both among, and within, 13 European and Asian countries at different levels of socioeconomic development. We found that cyberbullying was a diverse phenomenon that showed wide variations when it came to the overlap between cybervictimization and traditional victimization across countries. Our findings suggest that both traditional and cyberbullying should be considered within anti-bullying practices. Because these types of bullying overlap, interventions should focus on how to reduce bullying behavior, rather than primarily focus on where the bullying takes place [30]. However, greater cross-cultural understanding of the observed differences in victimization between the sexes, and the bullying context, would also help to enhance anti-bullying efforts. Bullying victimization needs to be recognized as a major risk for mental health. We found that being a victim of traditional bullying, and also experiencing cyberbullying, had a stronger association with psychopathology than just one form of bullying. That is an important consideration when planning anti-bullying intervention strategies. Although this study was observational, it supports the assumption that anti-bullying interventions may reduce victimization; implementing anti-bullying interventions, and studying their effectiveness also in developing countries, is important.

## Supplementary Information

Below is the link to the electronic supplementary material.

Supplementary file1 (PDF 409 KB)

## Declarations

### Software application

SAS 9.4 for Windows (SAS Institute Inc. Cary, NC, USA, 2012).

## References

[1] Gladden RM, Vivolo-Kantor AM, Hamburger ME, Lumpkin CD (2014) Bullying Surveillance Among Youths: Uniform Definitions for Public Health and Recommended Data Elements, Version 1.0. National Center for Injury Prevention and Control, Centers for Disease Control and Prevention and U.S. Department of Education, Atlanta, GA, USA, p. 7. https://www.cdc.gov/violenceprevention/pdf/bullying-definitions-final-a.pdf Accessed 3 Dec 2019

[2] Fleming LC, Jacobsen KH (2010). Bullying among middle-school students in low and middle income countries. Health Promot Int.

[3] Zaborskis A, Ilionsky G, Tesler R, Heinz A (2019). The association between cyberbullying, school bullying, and suicidality among adolescents. Crisis.

[4] Wolke D, Lee K, Guy A (2017). Cyberbullying: a storm in a teacup?. Eur Child Adolesc Psychiatry.

[5] Tiiri E, Luntamo T, Mishina K, Sillanmäki L, Brunstein Klomek A, Sourander A (2020). Did bullying victimization decrease after nationwide school-based anti-bullying program? A time-trend study. J Am Acad Child Adolesc Psychiatry.

[6] Athanasiou K, Melegkovits E, Andrie EK, Magoulas C, Tzavara CK, Richardson C, Greydanus D, Tsolia M, Tsitsika AK (2018). Cross-national aspects of cyberbullying victimization among 14–17-year-old adolescents across seven European countries. BMC Public Health.

[7] Tsitsika A, Janikian M, Wójcik S, Makaruk K, Tzavela E, Tzavara C, Greydanus D, Merrick J, Richardson C (2015). Cyberbullying victimization prevalence and associations with internalizing and externalizing problems among adolescents in six European countries. Comput Hum Behav.

[8] Brunstein Klomek A, Sourander A, Elonheimo H (2015). Bullying by peers in childhood and effects on psychopathology, suicidality, and criminality in adulthood. Lancet Psychiatry.

[9] Copeland WE, Wolke D, Angold A, Costello J (2013). Adult psychiatric outcomes of bullying and being bullied by peers in childhood and adolescence. JAMA Psychiat.

[10] Wolke D, Lereya ST (2015). Long-term effects of bullying. Arch Dis Child.

[11] Brunstein Klomek A, Sourander A, Niemelä S, Kumpulainen K, Piha J, Tamminen T, Almqvist F, Gould MS (2009). Childhood bullying behaviors as a risk for suicide attempts and completed suicides: a population-based birth cohort study. J Am Acad Child Adolesc Psychiatry.

[12] Takizawa R, Maughan B, Arseneault L (2014). Adult health outcomes of childhood bullying victimization: evidence from a five-decade longitudinal British birth cohort. Am J Psychiatry.

[13] Jantzer V, Schlander M, Hafner J, Parzer P, Trick S, Resch F, Kaess M (2019). The cost incurred by victims of bullying from a societal perspective: estimates based on a German online survey of adolescents. Eur Child Adolesc Psychiatry.

[14] Nansel TR, Craig W, Overpeck MD, Saluja G, Ruan WJ, the Health Behaviour in School-aged Children Bullying Analyses Working Group (2004). Cross-national consistency in the relationship between bullying behaviors and psychosocial adjustment. Arch Pediatr Adolesc Med.

[15] Due P, Merlo J, Harel-Fisch Y, Damsgaard MT, Holstein BE, Hetland J, Currie C, Nic Gabhainn S, Gaspar de Matos M, Lynch J (2009). Socioeconomic Inequality in exposure to bullying during adolescence: a comparative, cross-sectional, multilevel study in 35 countries. Am J Public Health.

[16] Due P, Holstein BE, Soc MS (2008). Bullying victimization among 13 to 15-year-old school children: results from two comparative studies in 66 countries and regions. Int J Adolesc Med Health.

[17] Due P, Holstein BE, Lynch J, Diderichsen F, Nic Gabhainn S, Scheidt P, Currie C, the Health Behaviour in School-Aged Children Bullying Working Group (2005). Bullying and symptoms among school-aged children: international comparative cross sectional study in 28 countries. Eur J Public Health.

[18] Elgar FJ, McKinnon B, Walsh SD, Freeman J, Donnelly PD, Gaspar de Matos M, Gariepy G, Aleman-Diaz AY, Pickett W, Molcho M, Currie C (2015). Structural determinants of youth bullying and fighting in 79 countries. J Adolesc Health.

[19] Nguyen A, Bradshaw C, Townsend L, Bass J (2020). Prevalence and correlates of bullying victimization in four low-resource countries. J Interpers Violence.

[20] Pengpid S, Peltzer K (2016) Parental Involvement, Health Behaviors and Mental Health among School-going Adolescents in Six Asian Countries. ASR Chiang Mai Univ J Soc Sci Hum 3:115−132. 10.12982/CMUJASR.2016.0007

[21] Lee YL, Kwon Y, Yang S, Park S, Kim EM, Na EY (2017). Differences in friendship networks and experiences of cyberbullying among korean and australian adolescents. J Genet Psychol.

[22] Cosma A, Walsh SD, Chester KL, Callaghan M, Molcho M, Craig W, Pickett W (2020). Bullying victimization: time trends and the overlap between traditional and cyberbullying across countries in Europe and North America. Int J Public Health.

[23] Görzig A, Milosevic T, Staksrud E (2017). Cyberbullying victimization in context: the role of social inequalities in countries and regions. J Cross Cult Psychol.

[24] Li Q (2008). A cross-cultural comparison of adolescents' experience related to cyberbullying. Educ Res.

[25] Ortega R, Elipe P, Mora-Merchán JA, Genta ML, Brighi A, Guarini A, Smith PK, Thompson F, Tippett N (2012). The emotional impact of bullying and cyberbullying on victims: a european cross-national study. Aggress Behav.

[26] Olweus D (2012). Invited expert discussion paper. Cyberbullying: an overrated phenomenon?. Eur J Dev Psychol.

[27] Hinduja S, Patchin JW (2012). Commentary. Cyberbullying: Neither an epidemic nor a rarity. Eur J Dev Psychol.

[28] Smith PK, Jimerson SR, Nickerson AB, Mayer MJ, Furlong MJ (2012). Cyberbullying and cyber aggression. Handbook of school violence and school safety: International research and practice.

[29] Yang S, Stewart R, Kim J, Kim S, Shin I, Dewey ME, Maskey S, Yoon J (2013). Differences in predictors of traditional and cyber-bullying: a 2-year longitudinal study in Korean school children. Eur Child Adolesc Psychiatry.

[30] Modecki KL, Minchin J, Harbaugh AG, Guerra NG, Runions KC (2014). Bullying prevalence across contexts: a meta-analysis measuring cyber and traditional bullying. J Adolesc Health.

[31] Sourander A, Chudal R, Skokauskas N, Al-Ansari AM, Brunstein Klomek A, Pornnoppadol C, Kolaitis G, Maezono J, Steinhausen HC, Slobodskaya H, Kaneko H, Regmee J, Li L, Nguyen MH, Grimland M, Osokina O, Ong SH, Praharaj SK, Lesinskienė S, Fossum S, Wiguna T, Makasheva VA, Lehti V (2018). Unmet needs of child and adolescent psychiatrists among Asian and European countries: Does the Human Development Index (HDI) count?. Eur Child Adolesc Psychiatry.

[32] Hamada S, Kaneko H, Ogura M, Yamawaki A, Maezono J, Sillanmäki L, Sourander A, Honjo S (2018). Association between bullying behavior, perceived school safety, and self-cutting: a Japanese population-based school survey. Child Adolesc Ment Health.

[33] Peng Z, Klomek AB, Li L, Su X, Sillanmäki L, Chudal R, Sourander A (2019). Associations between Chinese adolescents subjected to traditional and cyber bullying and suicidal ideation, self-harm and suicide attempts. BMC Psychiatry.

[34] Sourander A, Koskelainen M, Niemela S, Rihko M, Ristkari T, Lindroos J (2012). Changes in adolescents’ mental health and use of alcohol and tobacco: a 10-year time-trend study of Finnish adolescents. Eur Child Adolesc Psychiatry.

[35] Goodman R (2001). Psychometric properties of the strengths and difficulties questionnaire. J Am Acad Child Adolesc Psychiatry.

[36] Koskelainen M, Sourander A, Vauras M (2001). Self-reported strengths and difficulties in a community sample of Finnish adolescents. Eur Child Adolesc Psychiatry.

[37] Youth in Mind. Downloadable SDQs and related items. https://www.sdqinfo.org/py/sdqinfo/b0.py. Accessed 15 Feb 2021

[38] Goodman R, Renfrew D, Mullick M (2000). Predicting type of psychiatric disorder from Strengths and Difficulties Questionnaire (SDQ) scores in child mental health clinics in London and Dhaka. Eur Child Adolesc Psychiatry.

[39] Obel C, Heiervang E, Rodriguez A, Heyerdahl S, Smedje H, Sourander A, Guethmundsson OO, Clench-Aas J, Christensen E, Heian F, Mathiesen KS, Magnusson P, Njarethvik U, Koskelainen M, Ronning JA, Stormark KM, Olsen J (2004) The strengths and difficulties questionnaire in the nordic countries. Eur Child Adolesc Psychiatry 13(Suppl 2):II32–II39. 10.1007/s00787-004-2006-2 10.1007/s00787-004-2006-2 15243784

[40] Maezono J, Hamada S, Sillanmäki L, Kaneko H, Ogura M, Lempinen L, Sourander A (2019) Cross‐cultural, population‐based study on adolescent body image and eating distress in Japan and Finland. Scand J Psychol 60:67–76. 10.1111/sjop.12485 10.1111/sjop.12485 PMC7379298 30395688

[41] Goodman R (1997) The Strengths and Difficulties Questionnaire: a research note. J Child Psychol Psychiatry 38:581–586. 10.1111/j.1469-7610.1997.tb01545.x 10.1111/j.1469-7610.1997.tb01545.x 9255702

[42] Goodman A, Lamping, DL, Ploubidis, GB (2010) When to Use Broader Internalising and Externalising Subscales Instead of the Hypothesised Five Subscales on the Strengths and Difficulties Questionnaire (SDQ): Data from British Parents, Teachers and Children. J Abnorm Child Psychol 38:1179–1191. 10.1007/s10802-010-9434-x 10.1007/s10802-010-9434-x 20623175

[43] United Nations Development Programme (2016) Human Development Index. Human Development Reports. http://hdr.undp.org/en/content/human-development-index-hdi Accesssed 3 August 2018

[44] Smith PK (2014). Understanding school bullying: its nature and prevention strategies.

[45] Ybarra ML, Diener-West M, Leaf PJ (2007). Examining the overlap in internet harassment and school bullying: implications for school intervention. J Adolesc Health.

[46] Flygare E, Frånberg GM, Gill P, Johansson B, Lindberg O, Osbeck C, Söderström Å (2011) Evaluation of anti-bullying methods. Report 353. National Agency for Education, Stockholm, Sweden, pp 16–20. https://www.skolverket.se/getFile?file=2849 Accessed 30 Oct 2019

[47] Barzilay S, Brunstein Klomek A, Apter A, Carli V, Wasserman C, Hadlaczky G, Hoven CW, Sarchiapone M, Balazs J, Kereszteny A, Brunner R, Kaess M, Bobes J, Saiz P, Cosman D, Haring C, Banzer R, Corcoran P, Kahn JP, Postuvan V, Podlogar T, Sisask M, Varnik A, Wasserman D (2017). Bullying victimization and suicide ideation and behavior among adolescents in Europe: a 10-country study. J Adolesc Health.

[48] Smith PK, López-Castro L, Robinson S, Görzig A (2019). Consistency of gender differences in bullying in cross-cultural surveys. Aggress Violent Behav.

[49] Gaffney H, Ttofi MM, Farrington DP (2019). Evaluating the effectiveness of school-bullying prevention programs: an updated meta-analytical review. Aggress Violent Behav.

[50] Gaffney H, Farrington DP, Espelage DL, Ttofi MM (2019). Are cyberbullying intervention and prevention programs effective? A systematic and meta-analytical review. Aggress Violent Behav.

[51] Sivaraman B, Nye E, Bowes L (2019). School-based anti-bullying interventions for adolescents in low- and middle-income countries: a systematic review. Aggress Violent Behav.

[52] Kowalski RM, Giumetti GW, Schroeder AN, Lattanner MR (2014). Bullying in the digital age: a critical review and meta-analysis of cyberbullying research among youth. Psychol Bull.

[53] Reijntjes A, Kamphuis JH, Prinzie P, Telch MJ (2010). Peer victimization and internalizing problems in children: a meta-analysis of longitudinal studies. Child Abuse Negl.

[54] Reijntjes A, Kamphuis JH, Prinzie P, Boelen PA, van der Schoot M, Telch MJ (2011). Prospective linkages between peer victimization and externalizing problems in children: a meta-analysis. Aggress Behav.

[55] Sourander A, Jensen P, Rønning J, Niemelä S, Helenius H, Sillanmäki L, Kumpulainen K, Piha J, Tamminen T, Moilanen I, Almqvist F (2007). What is the early adulthood outcome of boys who bully or are bullied in childhood? The Finnish “From a Boy to a Man” study. Pediatrics.

[56] Sourander A, Gyllenberg D, Brunstein Klomek A, Sillanmäki L, Ilola AM, Kumpulainen K (2016). Association of bullying behavior at 8 years of age and use of specialized services for psychiatric disorders by 29 years of age. JAMA Psychiat.

[57] Arseneault L (2018). Annual research review: the persistent and pervasive impact of being bullied in childhood and adolescence: implications for policy and practice. J Child Psychol Psychiatry.

[58] Espelage D, Low S, Jimerson S (2014). Understanding school climate, aggression, peer victimization, and bully perpetration: contemporary science, practice, and policy. Sch Psychol Q.

[59] Elgar FJ, Pickett KE, Pickett W, Craig W, Molcho M, Hurrelmann K, Lenzi M (2013). School bullying, homicide and income inequality: a cross-national pooled time series analysis. Int J Public Health.

[60] Glasgow RE, Lichtenstein E, Marcus AC (2003). Why don't we see more translation of health promotion research to practice? Rethinking the efficacy-to-effectiveness transition. Am J Public Health.

[61] Zych I, Farrington DP, Ttofi MM (2019). Protective factors against bullying and cyberbullying: a systematic review of meta-analyses. Aggress Violent Behav.

[62] Wolke D, Tippett N, Dantchev S (2015). Bullying in the family: sibling bullying. Lancet Psychiatry.

[63] Andersen LP, Labriola M, Andersen JH, Lund T, Hansen CD (2015). Bullied at school, bullied at work: a prospective study. BMC Psychol.

[64] International Labour Organization, ILO, NORMLEX (1973) Ratifications of C138 - Minimum Age Convention, No. 138. https://www.ilo.org/dyn/normlex/en/f?p=1000:11300:0::NO:11300:P11300_INSTRUMENT_ID:312283 Accessed 5 Feb 2020

[65] International Labour Organization, ILO, NATLEX. Islamic Republic of Iran. Labour codes, general labour and employment acts. https://www.ilo.org/dyn/natlex/natlex4.detail?p_lang=en&p_isn=21843 Accessed 5 Feb 2020

