# Supplementary material for: Victimization by traditional bullying and cyberbullying and the combination of these among adolescents in 13 European and Asian countries
Source: Eur Child Adolesc Psychiatry. 2021 Apr 21;31(9):1391–404. doi: 10.1007/s00787-021-01779-6 (PMC9402766; doi:10.1007/s00787-021-01779-6)
Supplement: Supplementary file 1 — Supplementary file1 (PDF 409 KB) [file 787_2021_1779_MOESM1_ESM.pdf]

Victimization by traditional bullying and cyberbullying and the combination of these among adolescents in 13 European and Asian countries

European Child & Adolescent Psychiatry

Chudal Roshan*, Tiiri Elina*, Brunstein Klomek Anat, Ong Say How, Fossum Sturla, Kaneko Hitoshi, Kolaitis Gerasimos, Lesinskiene Sigita, Li Liping, Nguyen Huong Mai, Praharaj Samir Kumar, Sillanmäki Lauri, Slobodskaya Helena R, Srabstein Jorge C, Wiguna Tjhin, Zamani Zahra, Sourander Andre, the EACMHS Study Group

*Joint first authors

Corresponding author: Andre Sourander, andsou@utu.fi

Department of Child Psychiatry, University of Turku, Turku, Finland; Turku University Hospital, Turku, Finland and INVEST Research Flagship, University of Turku, Turku, Finland.

Assessment of the associations between victimization and the availability of anti-bullying interventions and the development of the country and within-country differences in victimization

Focused anti-bullying interventions

The child mental health (CAMH) experts who participated in the Eurasian Child Mental Health Study (EACMHS) provided information on the availability of anti-bullying interventions in their country. The countries were categorized as having anti-bullying interventions if bullying was regarded as a national priority and anti-bullying interventions were available at the participating schools before the survey was conducted. Four countries provided focused anti-bullying interventions. These were Finland, Greece, Lithuania and Norway. The CAMH experts in these countries provided further information on the interventions that were available. Many interventions included both universal school-based actions, which aimed to prevent bullying, and indicated actions like restorative practices or discussions with those involved. Some of the programs included anti-cyberbullying components. The interventions included the Finnish KiVa program [1], the Norwegian Zero program [2] and the Norwegian Olweus Bullying Prevention Program, (OBPP) [3]. In Lithuania, every school implements at least one anti-bullying program including the OBPP. Several anti-bullying programs have been implemented in Greece [4]. In some countries, such as Israel and Singapore, there have been school-based programs that included bullying but did not solely focus on it. These were not considered to be focused anti-bullying programs.

The **KiVa program** is a research-based, whole-school anti-bullying intervention, which conceptualizes bullying as a group phenomenon. It aims to make positive changes, by encouraging bystanders to support victims and not tolerate bullying. This reduces the bullies' motivation to bully. Universal, preventive, actions are provided for all students, including lessons or themes and a virtual learning environment. Indicated actions include discussions for those involved in bullying and separate discussions with potential

supporters of the victim [1]. Studies have reported that KiVa reduced traditional victimization [5], especially among younger students [1,6], and had beneficial effects on cyberbullying [1,7].

The **Zero program** is based on understanding that bullying is mainly proactive aggression and that if teachers exert weak control and support in classes this may lead to weak social cohesion. This, in turn, may provide a climate for bullying [2,8]. Zero aims to create a safe school environment that prevents aggression and emphasizes competent classroom management and the responsibilities of adults. Preventive actions include direct work in the classroom, that focus on bullying, but also improve empathy among students. Indicated actions include discussions with victims, and later, with bullies. Teachers also contact the parents of those involved. Zero has been found to reduce victimization, especially among younger students [2].

The **OBPP** is the oldest anti-bullying intervention and it dates back to the 1980s [3]. It is a whole-school program that focuses on making positive changes in the school environment, in order to prevent, and reduce, bullying and achieve better peer relationships. The OBPP builds on four principles that emphasize the responsibility of adults. They are expected to show a real interest in students and provide authoritative and positive role models. Teachers and other adults should also set firm limits about what is unacceptable behavior. They are also expected to respond positively to appropriate behavior and implement non-physical, non-hostile action when rules are broken. The OBPP includes school-wide, classroom, individual and community level components. It focuses on prevention, but also includes indicated actions for those students who have been involved in bullying. The OBPP has been widely studied and has been repeatedly found to be effective in reducing bullying, especially in Norway [3,5,9]. Stronger positive findings have been reported for younger students [9].

Statistical methods

A composite variable was used to estimate the odds ratios (ORs) using the generalized estimating equation (GEE) models. The countries were grouped based on their Human Development Index (HDI) ranks into two categories: very high HDI (ranks 1–51) and high/medium HDI (52–147). These were based on the 2016 ranks provided by the United Nations Development Programme [10]. The very high HDI category comprised eight countries: Norway (HDI rank 1), Singapore (5), Japan (17), Israel (19), Finland (23), Greece (29), Lithuania (37) and Russia (49). The high/medium category comprised five countries: Iran (69), China (90), Indonesia (113), Vietnam (115) and India (131). The 13 countries were also grouped by whether anti-bullying interventions were available in their schools. These two factors were then combined into three composite variables. There were eight very high HDI countries: four with anti-bullying programs (Finland, Greece, Lithuania and Norway) and four without such programs (Israel, Japan, Russia and Singapore). There were also five high/medium HDI countries with no programs (China, India, Indonesia, Iran and Vietnam). The ORs were estimated using equal weights for each country, so that they all played an equal role in the analyses, regardless of their sample size.

Variations between the schools and between the countries were estimated. To assess variations between the schools, model-generated, age-adjusted predicted probabilities for any victimization were estimated by sex for each school in each country. If a school had less than ten girls or boys who were victimized, the school was excluded from the analyses regarding this sex.

Two-sided *p*-values of less than 0.05 were considered statistically significant. The statistical analyses were conducted using SAS 9.4 for Windows (SAS Institute Inc. Cary, NC, USA, 2012).

References

1. Salmivalli C, Kärnä A, Poskiparta E (2011) Counteracting bullying in Finland: The KiVa program and its effects on different forms of being bullied. Int J Behav Dev 35:405–411. https://doi.org/10.1177/0165025411407457
2. Roland E, Bru E, Midthassel UV, Vaaland GS (2010) The Zero programme against bullying: effects of the programme in the context of the Norwegian manifesto against bullying. Soc Psychol Educ 13:41–55. https://doi.org/10.1007/s11218-009-9096-0
3. Olweus D, Limber S (2010) Bullying in school: Evaluation and dissemination of the Olweus bullying prevention program. Am J Orthopsychiat 80:124–134. https://doi.org/10.1111/j.1939-0025.2010.01015.x
4. Artinopoulou V, Michael I (2014) Good anti-bullying practices in partnership countries: Greece. In: Artinopoulou V, Michael I (eds) European Guide of Anti-bullying Good Practices. European Anti-Bullying Network (EAN). A Project funded by EU Daphne III Programme, pp.13–14. https://www.academia.edu/9670211/Artinopoulou_V._and_Michael_I._Eds_2014_._European_Guide_on_Antibullying_Good_Practices Accessed 13 June 2019
5. Gaffney H, Farrington DP, Ttofi MM (2019) Examining the Effectiveness of School-Bullying Intervention Programs Globally: a Meta-analysis. Int J Bullying Prev 1:14–31. https://doi.org/10.1007/s42380-019-0007-4
6. Kärnä A, Voeten M, Little TD, Alanen E, Poskiparta E, Salmivalli C (2013) Effectiveness of the KiVa Antibullying Program: Grades 1–3 and 7–9. J Educ Psychol 105:535–551. https://doi.org/10.1037/a0030417
7. Williford A, Elledge LC, Boulton AJ, DePaolis KJ, Little TD, Salmivalli C (2013) Effects of the KiVa antibullying program on cyberbullying and cybervictimization frequency among Finnish youth. J Clin Child Adolesc Psychol 42:820–833. https://doi.org/10.1080/15374416.2013.787623
8. Roland E, Midthassel UV (2012) The Zero program. New Dir Youth Dev 133:29–39. https://doi.org/10.1002/yd.20005
9. Limber SP, Olweus D, Wang W, Masiello M, Breivik K (2018) Evaluation of the Olweus Bullying Prevention Program: A large scale study of U.S. students in grades 3–11. J School Psychol 69;56–72. https://doi.org/10.1016/j.jsp.2018.04.004
10. United Nations Development Programme (2016) Human Development Index. Human Development Reports. http://hdr.undp.org/en/content/human-development-index-hdi Accesssed 3 August 2018

Table S1 Victimization by any bullying of girls and boys by frequency and country. Any victimization refers to exposure to either traditional bullying, cyberbullying or the combination of these.

	Girls				Boys			
	Never *n* (%)	Less than once a week *n* (%)	More than once a week *n* (%)	Almost every day *n* (%)	Never *n* (%)	Less than once a week *n* (%)	More than once a week *n* (%)	Almost every day *n* (%)
Japan	784 (84.0)	86 (9.2)	40 (4.3)	23 (2.5)	725 (83.7)	79 (9.1)	41 (4.7)	21 (2.4)
Greece	475 (85.4)	65 (11.7)	9 (1.6)	7 (1.3)	395 (81.8)	67 (13.9)	10 (2.1)	11 (2.3)
Norway	773 (77.5)	159 (16.8)	33 (3.5)	21 (2.2)	791 (82.9)	106 (11.1)	30 (3.1)	27 (2.8)
China	844 (81.7)	136 (13.2)	36 (3.5)	17 (1.7)	781 (74.0)	206 (19.5)	45 (4.3)	24 (2.3)
India	706 (82.9)	100 (11.7)	17 (2.0)	29 (3.4)	564 (70.9)	156 (19.6)	36 (4.5)	40 (5.0)
Finland	1,060 (71.7)	298 (20.2)	88 (6.0)	33 (2.2)	1,055 (73.5)	294 (20.5)	68 (4.7)	19 (1.3)
Singapore	794 (72.1)	223 (20.2)	54 (4.9)	31 (2.8)	744 (70.2)	214 (20.2)	63 (5.9)	39 (3.7)
Vietnam	359 (74.2)	97 (20.0)	23 (4.8)	5 (1.0)	297 (64.3)	111 (24.0)	36 (7.8)	18 (3.9)
Israel	469 (67.2)	160 (22.9)	54 (7.7)	15 (2.2)	357 (61.7)	169 (29.2)	38 (6.6)	15 (2.6)
Iran	395 (70.9)	114 (20.5)	25 (4.5)	23 (4.1)	359 (57.8)	171 (27.5)	51 (8.2)	40 (6.4)
Lithuania	783 (62.6)	318 (25.4)	90 (7.2)	59 (4.7)	790 (63.4)	300 (24.1)	70 (5.6)	87 (7.0)
Russia	343 (62.8)	152 (27.8)	34 (6.2)	17 (3.1)	300 (60.4)	106 (21.3)	40 (8.1)	51 (10.3)
Indonesia	312 (57.6)	140 (25.8)	54 (10.0)	36 (6.6)	261 (54.3)	123 (25.6)	54 (11.2)	43 (8.9)
Total	8,057 (73.4)	2,048 (18.7)	557 (5.1)	316 (2.9)	7,419 (70.4)	2,102 (20.0)	582 (5.5)	435 (4.1)

Note: The limited number of cases in some categories did not allow further analyses of the frequency of bullying.

Table S2 Traditional victimization only, cyberbullying victimization only and combined victimization of girls by country

Country	Total *n*	Traditional only *n* (%)	Cyber only *n* (%)	Combined *n* (%)	Traditional only OR (95 %CI)	Cyber only OR (95 %CI)	Combined OR (95 %CI)
Japan	922	127 (13.8)	10 (1.1)	9 (1.0)	1	1	1
Greece	555	54 (9.7)	15 (2.7)	11 (2.0)	0.71 (0.47−1.08)	**2.52 (1.07−5.97)**	1.86 (0.82−4.20)
Norway	946	64 (6.8)	80 (8.5)	69 (7.3)	**0.54 (0.35−0.82)**	**8.27 (3.47−19.68)**	**8.13 (4.35−15.21)**
China	1007	105 (10.4)	49 (4.9)	30 (3.0)	0.81 (0.56−1.17)	**4.61 (1.97−10.76)**	**2.92 (1.58−5.40)**
India	799	126 (15.8)	7 (0.9)	9 (1.1)	1.14 (0.62−2.06)	0.85 (0.27−2.68)	1.16 (0.48−2.79)
Finland	1467	251 (17.1)	63 (4.3)	101 (6.9)	**1.56 (1.09−2.23)**	**4.44 (1.95−10.10)**	**8.22 (4.52−14.94)**
Singapore	1102	144 (13.1)	44 (4.0)	120 (10.9)	1.14 (0.77−1.67)	**4.20 (1.80−9.77)**	**12.59 (6.51−24.36)**
Vietnam	483	97 (20.1)	13 (2.7)	15 (3.1)	**1.64 (1.16−2.34)**	2.82 (0.98−8.11)	**3.46 (1.90−6.32)**
Israel	687	120 (17.5)	51 (7.4)	53 (7.7)	1.47 (0.97−2.21)	**8.70 (3.84−19.71)**	**7.90 (3.58−17.43)**
Iran	526	67 (12.7)	60 (11.4)	28 (5.3)	1.15 (0.75−1.75)	**12.30 (5.17−29.27)**	**6.37 (3.06−13.27)**
Lithuania	1209	314 (26.0)	64 (5.3)	76 (6.3)	**2.76 (1.89−4.03)**	**6.39 (2.77−14.74)**	**8.63 (4.77−15.63)**
Russia	542	106 (19.6)	44 (8.1)	52 (9.6)	**2.02 (1.37−2.99)**	**9.69 (4.09−22.92)**	**13.09 (7.07−24.21)**
Indonesia	542	111 (20.5)	38 (7.0)	81 (14.9)	**2.30 (1.37−3.87)**	**9.81 (3.87−24.85)**	**21.33 (9.65−47.17)**

Note: GEE model with school-wise clusters included. Adjusted for age. Differences in the numbers of participants between tables are due to missing information. Bold type indicates statistical significance of at least $p < 0.05$. *OR* odds ratio.

Table S3 Traditional victimization only, cyberbullying victimization only and combined victimization of boys by country

Country	Total *n*	Traditional only *n* (%)	Cyber only *n* (%)	Combined *n* (%)	Traditional only OR (95 %CI)	Cyber only OR (95 %CI)	Combined OR (95 %CI)
Japan	843	118 (14.0)	7 (0.8)	13 (1.5)	1	1	1
Greece	482	65 (13.5)	13 (2.7)	10 (2.1)	0.98 (0.63−1.52)	**3.33 (1.33−8.30)**	1.47 (0.43−5.01)
Norway	954	79 (8.3)	43 (4.5)	41 (4.3)	**0.62 (0.44−0.87)**	**5.19 (2.48−10.85)**	**3.34 (1.59−7.00)**
China	1025	153 (14.9)	67 (6.5)	40 (3.9)	1.20 (0.87−1.66)	**8.52 (4.29−16.95)**	**3.65 (1.80−7.42)**
India	727	175 (24.1)	12 (1.7)	25 (3.4)	**2.24 (1.43−3.52)**	2.32 (0.85−6.33)	**3.23 (1.24−8.40)**
Finland	1422	293 (20.6)	37 (2.6)	47 (3.3)	**1.87 (1.41−2.47)**	**3.36 (1.64−6.89)**	**3.17 (1.56−6.44)**
Singapore	1055	202 (19.2)	39 (3.7)	72 (6.8)	**1.76 (1.32−2.34)**	**5.02 (2.40−10.51)**	**6.83 (3.49−13.36)**
Vietnam	462	123 (26.6)	13 (2.8)	29 (6.3)	**2.71 (1.71−4.32)**	**4.24 (2.11−8.52)**	**6.80 (3.27−14.16)**
Israel	570	138 (24.2)	27 (4.7)	54 (9.5)	**2.89 (1.76−2.98)**	**6.34 (3.08−13.06)**	**10.43 (4.89−22.23)**
Iran	620	147 (23.7)	46 (7.4)	68 (11.0)	**2.82 (2.14−3.72)**	**11.78 (5.66−24.51)**	**13.51 (6.94−26.30)**
Lithuania	1171	302 (25.8)	46 (3.9)	82 (7.0)	**2.73 (2.04−3.64)**	**5.77 (2.76−12.04)**	**7.47 (3.40−16.40)**
Russia	479	75 (15.7)	82 (17.1)	39 (8.1)	**1.74 (1.14−2.66)**	**27.65 (12.51−61.10)**	**9.59 (4.49−20.48)**
Indonesia	481	130 (27.0)	29 (6.0)	61 (12.7)	**3.38 (2.08−5.49)**	**10.90 (5.11−23.24)**	**18.39 (6.63−51.02)**

Note: GEE model with school-wise clusters included. Adjusted for age. Differences in the numbers of participants between tables are due to missing information. Bold type indicates statistical significance of at least $p < 0.05$. *OR* odds ratio.

Fig. S1 Predicted probabilities of any bullying victimization by school and country, adjusted for age. The X axis shows schools in random order. Cases were excluded in schools with less than ten girls or boys.

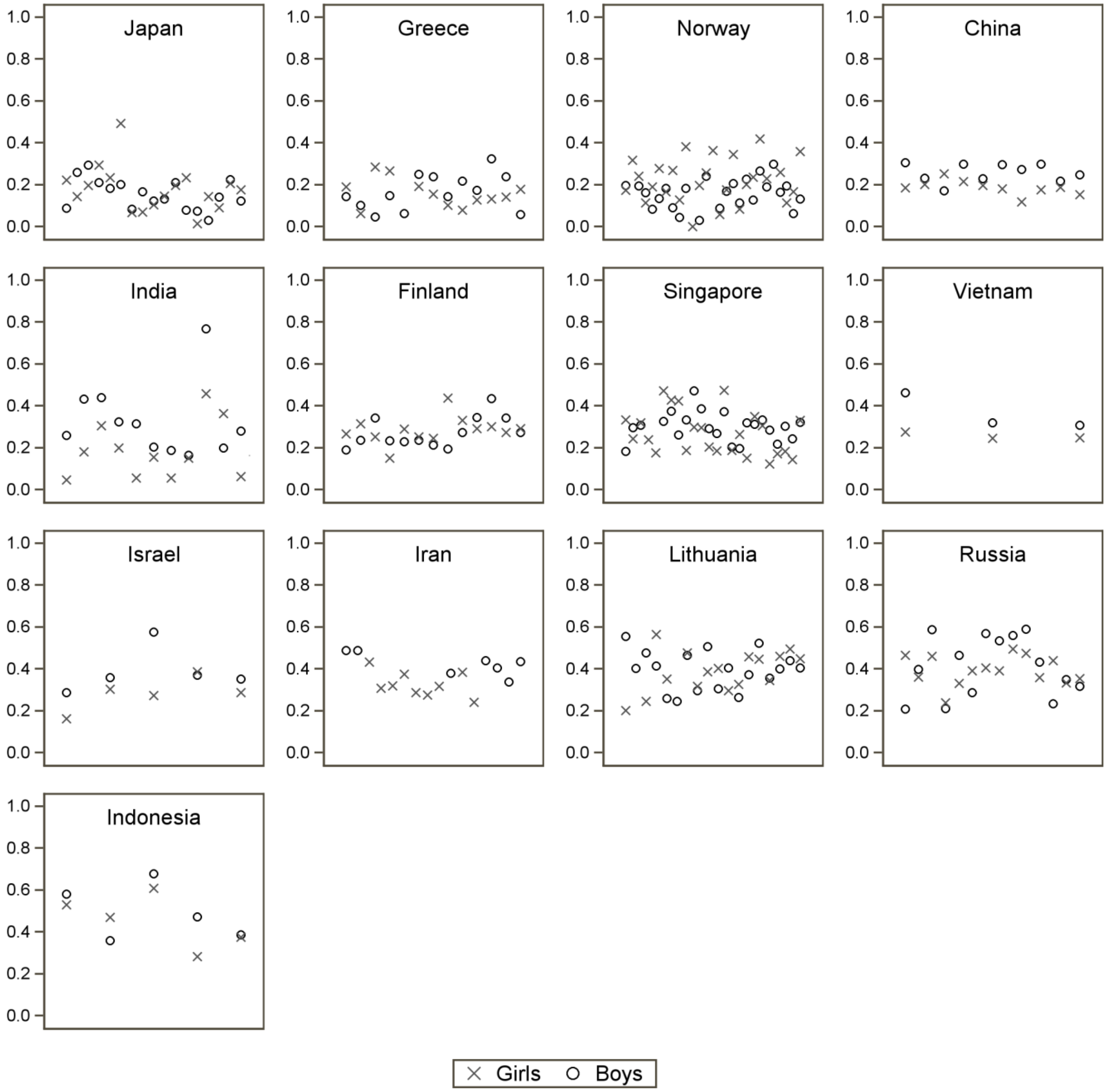

Table S4 Comparison between the Human Development Index classification and anti-bullying interventions in relation to traditional victimization only, cyberbullying victimization only and combined victimization of girls and boys

	Girls			Boys		
	Traditional only OR (95% CI)	Cyber only OR (95% CI)	Combined OR (95% CI)	Traditional only OR (95% CI)	Cyber only OR (95% CI)	Combined OR (95% CI)
Very high HDI and no intervention vs. very high HDI and intervention	1.13 (0.99−1.29)	1.09 (0.87−1.36)	**1.36 (1.12−1.65)**	**1.21 (1.06−1.39)**	**2.10 (1.65−2.68)**	**1.77 (1.42−2.22)**
High/medium HDI and no intervention vs. very high HDI and intervention	1.10 (0.96−1.25)	1.07 (0.86−1.32)	1.01 (0.83−1.24)	**1.61 (1.43−1.82)**	**1.68 (1.31−2.15)**	**2.09 (1.69−2.58)**
High/medium HDI and no intervention vs. very high HDI and no intervention	0.97 (0.84−1.12)	0.98 (0.77−1.24)	**0.75 (0.60−0.92)**	**1.33 (1.16−1.52)**	0.80 (0.63−1.01)	1.18 (0.95−1.46)

Note: Logistic regression models with equalized country weight and school-wise clusters included. Bold type indicates statistical significance of at least $p < 0.05$. *HDI* Human Development Index, *Intervention* anti-bullying intervention, *OR* odds ratio.
